# Thank you to *The Lancet Regional Health - Western Pacific'*s clinical and statistical peer reviewers in 2022

**DOI:** 10.1016/j.lanwpc.2023.100703

**Published:** 2023-01-31

Mohammed Aal

Peter Abad

Salim Abdool Karim

Fareed Abdullah

Seigo Abiru

Ananta Addala

Demilade Adedinsewo

Brendan Adkinson

Annabel Ahuriri-Driscoll

Jun Aida

Masaharu Akao

Matthew Akiyama

Naomi Akiyama

Murat Akova

Atalay Alem

Giuseppe Aleo

Karel Allegaert

Saif Al-Shamsi

Cleo Anastassopoulou

Craig Anderson

Yuichi Ando

Russell Andrews

Nasikarn Angkasekwinai

Khatia Antia

Md Asaduzzaman

Yoshimasa Aso

Masanori Atsukawa

Monique D Auger

Aviane Auguste

Asa Auta

Juan Carlos Ayus

Sina Azadnajafabad

Louise Baandrup

Shasha Bai

Anna Baiges

Karin Bammann

Zhikang Bao

Francisco Barrera

Rosana Barros

Larissa Bartlett

Romain Basmaci

Daniela Basso

Ariunzaya Bat-Erdene

Luc Bauchet

Sina Bavari

Ben Beaglehole

Julio Benavides

Yoav S Bergman

Marc Besselink

Giuseppe Biondi-Zoccai

Stephen Birch

Antony Black

Tony Blakely

Frank Bloomfield

Edimar Bocchi

Michael Boissonneault

Geremia B Bolli

Jennifer Lynn Bragg-Gresham

Paulo Braz-Silva

Eric Brunner

Lin Lin Bu

Eka Buadromo

Charlotte Buckley

Matthew Budoff

Ha Bui

My Hanh Bui

Dario Buioni

L Maximilian Buja

Gilbert Burckart

Rachael Burke

Jun Cai

Sarah Callinan

Michael Cameron

Jackie Campbell

Jonathon Campbell

Gianluca Campo

Nicole Campos

Yin Cao

Claudia Cardoso

Ben Carter

Thaddeus Carvajal

Susan Cassels

Robert Casson

Deniz Ceylan

Nina Chad

Shelly Chadha

Renoh Chalakkal

Timothy Chambers

Wing Bun Chan

Ellen T Chang

Jer Ming Chang

Sara Charleer

Marcia Chaves

Fangyao Chen

Fei Chen

Hua Chen

Jianjiu Chen

Renjie Chen

Runsen Chen

Simiao Chen

Tianmu Chen

Wei Chen

Wen Chen

Yingtai Chen

Yunbo Chen

Zhuo Chen

Vincent Chi-Chung Cheng

Wenwen Cheng

Yu Cheng

Carol Cheung

Isabel Chinen

Cheng-Hsun Chiu

Nai Rui Chng

Kenneth Chok

Ka Chun Chong

Om Choudhary

Mirjam Christ-Crain

Lingzhi Chu

Alvin Qijia Chua

Melvin Chua

Rungtip Chuanchuen

Michael L Chuang

Brian Chung

Nicola Cirillo

Jerome Visperas Cleofas

Gary Clifford

Tammy Clifford

Deirdre Collins

Alex Cook

Graham Cooke

Carol Coupland

Benjamin Cowling

Judith Crockett

Ginbert Cuaton

César Cuevas-Lara

Gabriel Culbert

Evan Cunningham

Ruth Cunningham

Tony Cunningham

Amarjargal Dagvadorj

Davaalkham Dambadarjaa

Bibhuti Bhusan Das

Adrian Davis

Christian De Geyter

Nicholas de Klerk

Manarangi De Silva

Edward Dee

Gilles Defraene

Michael Desjardins

Mohammed Ali Dheyab

Kumud Dhital

Bridget Dicker

Alexander Dilthey

Ding Ding

Jacqueline Dinnes

Tinh Doan

Matthew Dodd

Richard Dodel

Jianzeng Dong

E Ray Dorsey

Dar Dowlatshahi

Ugonma Dozie

Yiqi Du

Clemence Due

Tuyen Van Duong

Geoffrey Dusheiko

Bernard Ebruke

Christine Eckert

Alexandra Edelman

Eduardo Edelman Saul

Ryan Edwards

Mohamed Hassan Elnaem

Ahmed F El-Yazbi

Fantaye Endashawu

Akira Endo

Takayuki Enomoto

Oscar Espinosa

Davor Eterovic

Paolo Eusebi

Oluwaseun Falade-Nwulia

Michael Falster

Shangrong Fan

Ya Fang

Eric Faragher

Abdulaziz Farooq

Forough Farrokhyar

Luzhao Feng

Quishi Feng

Angeline Ferdinand

Pietro Ferrara

Giovanni Ferreira

Thibault Fiolet

Joanne Flavel

Joël Fokom Domgue

Fredrik Folke

Andrew Fox-Lewis

Anna Freni Sterrantino

Mingzhou Fu

Masashi Fujino

Noritoshi Fukushima

M Emmanuel Gabriel

Olivier Galy

Lakshmi Ganapathi

Kurubaran Ganasegeran

Mihir Gandhi

Sumanth Gandra

Mohd Ashraf Ganie

Qian Gao

Wei Gao

Wenjing Gao

Yanhang Gao

Marcial García

Monia Garofolo

Mario Gaudino

Serge Gauthier

Sergey Gautier

Michael Gensheimer

Thomas L Gentles

Jemma Geoghegan

Marco Geraci

Adrian Gheorghe

Laxmi Ghimire

Stuart Gilmour

Pere Ginès

Fabio Girardi

Deborah Gleeson

Brian Godman

Michael Goggins

Felicity Goodyear-Smith

Robert Gottlieb

Jiangtao Gou

Laksmi Govindasamy

Mario Gramegna

Ben Gray

Glenda E Gray

Kim Greaves

Jiafeng Gu

Liding Guan

Xiaodong Guan

Raphael Guimarães

David Gunnell

Chengxian Guo

Yutao Guo

Snehil Gupta

Robert Guthrie

Hideharu Hagiya

Omar Hahad

Joerg Haier

Brian Hall

Michaela Hall

Kaiyi Han

Yaling Han

Jordan R Hansford

Sally Hargreaves

Michael Harhay

Anne Hause

Anne M Hause

Daihai He

Pengcheng He

Ping He

Yulei He

Michael Hengartner

JoonNyung Heo

Jeffrey Hii

Nadine Hillock

Matt Hitchings

Mario Hlevnjak

Roger Ho

Zheng Jie Marc Ho

Johannes S M Hobbelen

Matthew Hobbs

Nicolas Hoertel

Harald Hoffmann

Hans Hogerzeil

Nusrat Homaira

Hitoshi Honda

Benjamin Horne

Katsuyuki Hotta

Jian Hou

Charlotte Höybye

Guoqing Hu

Shao-hua Hu

Yizhen Hu

Ganlin Huang

Pintong Huang

Yueqin Huang

Gesche Margarethe Huebner

David Hui

Edwin Hui

Neil Humphrey

Chien-Ching Hung

Chi-Sheng Hung

Ivan F N Hung

Jeong-kye Hwang

Zoë Hyde

Ross Iles

Wilawan Inchamnan

Kosuke Inoue

Nicola Irwin

Chieko Ishiguro

Miho Ishimaru

Miho Ishimaru

Masao Iwagami

Marie-Joelle Jabagi

Christopher Jackson

Henning Jacobsen

Dr Jagnoor Jagnoor

Yasmin Jahan

Mohammad Jalali

Rajiv Jalan

John Ji

John S Ji

Peng Jia

Xiaojiong Jia

Kaijun Jiang

Quanbao Jiang

Jeremy Johnson

Kai Jonas

Jovan Julien

Steven Julious

Jisun Jung

Lydia Sandrah Kaforau

Joseph Keawe‘aimoku Kaholokula

Kentaro Kamiya

Christiana Kartsonaki

Judith Katzenellenbogen

Ashish Kc

Chaofu Ke

Yang Ke

Karen Keddy

Roanne Keeton

Roya Kelishadi

John Kellett

Michelle Kelly

Mark Kelson

Zoe Kelson

Jonathan Kennedy

Tara Kessaram

Jim S Khan

Narges Khanjani

Ali Khashan

Resham Khatri

Stefan Kiechl

Vu Duy Kien

Ah-young Kim

Beom Kyung Kim

Yoonhee Kim

Tomomi Kimura

Paula King

Karin Kirchgatter

Yurie Kobashi

Masatake Kobayashi

Sarah Kobayashi

Satoshi Kodama

Katherine Koh

Shinsuke Koike

Takamasa Komiyama

Michelle Kondo

Yuanyuan Kong

Evangelos Kontopantelis

Thomas Kovesi

Yu Koyama

Fiorella Krapp

Evangelos Kritsotakis

Yi-Qun Kuang

Amit A Kulkarni

Jogender Kumar

Kenya Kusunose

Jelena Kuvač Kraljević

Min Ji Kwak

Sham Lal

Tea Lallukka

Bonnie Lam

Karin Landerl

Chris Lane

Frieder R Lang

Savita Lasrado

Amir Lass

Kate Laver

Huu Nhat Minh Le

Chaiwoo Lee

Heeyoung Lee

Jong-hyuk Lee

Kun Yun Lee

Victor Lee

Yen-Han Lee

Yew Kong Lee

Yong-ho Lee

Yiting Lei

Tomás León

Juan Leonardia

Stuart Leske

Janni Leung

Thomas Leung

David Levy

Nathaniel Lewis

Dongmin Li

Guangwei Li

Jia Li

Jian-Jun Li

Li Li

Li Li

Li Li

Ming Li

Qihan Li

Quanlei Li

Wen-Qing Li

Xi Li

Xia Li

Xiaohong Li

Xin Li

Yanguang Li

Ye Li

Zengbin Li

Yajun Liang

Hsunhsiang Liao

Francesco Licciardi

Cherry Lim

Seng Gee Lim

Chung-Ying Lin

Qian Lin

Tracy Lin

Benjamin Paul Linas

Xu Lingzhong

Bette Liu

Chaojie Liu

Cong Liu

Jue Liu

Lihong Liu

Miaomiao Liu

Min Liu

Weizhi Liu

Xiaodi Liu

Xiaoxue Liu

Xiwang Liu

Xuhui Liu

Zhongchun Liu

Jessica Lo

Patrícia Loggetto

Qian Long

Yicheng Long

Jiapeng Lu

Lin Lu

Tao Lu

Gregory Lucas

Lars Christian Lund

Tom Lung

Zeyu Luo

Paula Luz

Reidar Lystad

Wei Ma

Wenjun Ma

Evelyn MacDonald

Ricardo Machado Xavier

Charlotte Maggen

Christopher Maher

Ozayr Mahomed

Bernardo Mainou

Hei Wan Mak

Kanika Malik

Sandip Mandal

Sarah Maness

Anand Manoharan

Wenhui Mao

Xiaoyun Mao

Eloi Marijon

Michael Marks

Charles Marshall

Mary Faith Marshall

Christopher J Martin

Elizabeth K Martin

Gonzalo Martínez-Alés

Yusuf Ari Mashuri

Sérgio Massora

Prashant Mathur

Kousaku Matsubara

Ryozo Matsuda

Keitaro Matsuo

Fiona Matthews

Etuini Ma'u

Janya McCalman

Judith McCool

David McDaid

Emily McDonald

Allison M McFall

Stephen McGarvey

Mikhail Menis

Fiona Mensah

Gabriele Meyer

Gareth Meyer

Raanan Meyer

Jo Middleton

Philippa Middleton

Iona Millwood

Shiva Mishra

Rob Mitchell

Ana Mocumbi

Payal Modi

Shinichiro Morioka

John Bwalya Muma

David Murdoch

Shoji Nagao

Tomohito Nagaoka

Toshio Naito

Ronza Najjar Debbiny

Keiko Nakamura

Toshihiro Nanki

Keisuke Nansai

Camille Nebeker

Ian Neethling

Reuben Ng

Son Nghiem

Linh Ngo

Phuong Nguyen

Thu-Anh Nguyen

Tuan Nguyen

Michael Ni

Vahid M Nik

Mehru Nisha

Nobuo Nishi

Hiroshi Nishiura

Hiroyuki Noda

Shuhei Nomura

Peter Nordström

Anna Norhammar

John Norrie

Thomas Norris

Rachel Nugent

Milawaty Nurjono

Rónán O'Caoimh

Minsu Ock

John Oetzel

Ayodele Ogunleye

Motohiro Okada

KayLoni Olson

Isaac Olufadewa

Jason Ong

Tomiko Oskotsky

Kimberley O'Sullivan

Fadar Otitef

Chun-Quan Ou

Nonglak Pagaiya

Lorenzo Paglione

Christopher Palmer

Jay Pan

Monica Panca

Davide Papola

Noman Paracha

Wan Beom Park

Maria Parker

Marty Parker

Arvind Patel

Dimitrios Patoulias

Patricia Pavlinac

Deborah Payne

Michael Payne

Jon Pedersen

Sarah Pendlebury

Jose Perez-Molina

Julia Pescarini

Patrizio Pezzotti

Quang Pham

Angsana Phuphuakrat

Guglielmo Niccolò Piozzi

Nitesh Kumar Poddar

Wiphawadee Potisopha

Kumar Prabhash

Robin Prescott

Massimo Primignani

Manju Purohit

Fei Qi

Xingshun Qi

Menbao Qian

Jie Qiao

Gang Qin

Chao Qiu

Terence Quinn

Antonio Quispe

Motiur Rahman

Muhammad Aziz Rahman

Masna Rai

Taufiek Konrad Rajab

Leelani Rajapaksa

Singh Rajender

Kollengode Ramanathan

Ramya Ramaswami

Jose-Manuel Ramos-Rincon

Jonas Ranstam

Sergio Raposeiras Roubin

Seyed Vahid Razavi-Termeh

Michelle Redman-MacLaren

Judith Regensteiner

Mark Reger

Edna Maria Vissoci Reiche

Johan Reutfors

Taeho Greg Rhee

Henry Rice

Alan S Rigby

Thanitsara Rittiphairoj

Fiona Robards

Paul Robertson

Ilia Rochlin

Natalia Rodriguez

Kris Rogers

Amihai Rottenstreich

Jorge Gabriel Ruiz-Sánchez

Sukhyun Ryu

Banshi D Saboo

Kenji Sadamatsu

Manish Sadarangani

Sy Atezaz Saeed

Marc Saez

Manoj Kumar Sahu

Chan Hang Saing

Kenichi Sakakura

Zikria Saleem

I-Ching Sam

Mindy Sampson

Fabian Sanchis-Gomar

Rengaswamy Sankaranarayanan

S M Sapuan

Rajiv Saran

Iván Sarmiento

Simone Savastano

Robin Schaefer

Simone Scheithauer

Elena Schmidt

Felipe Schuch

Rosalie Schultz

Sandra Schüssler

Carlos Seas

Jwa Seungchik

Eithne Sexton

Jesmin Shafiq

Hong Shang

Deepak Sharma

Ye Shen

Sanjay Shete

Han-Ping Shi

Jian Shi

Leming Shi

Xiaoming Shi

Yaojiang Shi

Rahul Shidhaye

Yusuke Shimakawa

Jaimie Shing

Hiroki Shiomi

Nathan Shlobin

Colin Simpson

Gianfranco Sinagra

Balbir Bagicha Singh

Kuljit Singh

Laura Singh

Ravi Shankar Singh

Christoph Sinning

Bhawna Sirohi

Sudha Sivaram

S Sivasankaran

Nina Skjæret-Maroni

Helen Slater

Chris Smith

Margaret J Snowling

Samuel So

Winnie So

Hana Maizuliana Solehan

Kambiz Soltaninejad

Ranran Song

Yi Song

Jeffrey Sparks

Matthew Spittal

Mamidipalli Sai Spoorthy

Chandrashekhar Sreeramareddy

Ian Stanley

Zornitza Stark

Annalee Stearne

Nikolaos Stilianakis

Jack Stone

Yingying Su

Bo Sun

Gang Sun

Jing Sun

Li-Ying Sun

Pengming Sun

Qiang Sun

Hyuna Sung

Subapriya Suppiah

George Syrogiannopoulos

Rabih Tabet

Soichi Takeishi

Victor Talisa

Li Peng Tan

Melisa Mei Jin Tan

Myles Joshua Tan

Xiao Tan

Junko Tanaka

Kazuhiro Tanaka

Weiming Tang

Mary Anne Teariki

Hadi Tehrani

Weiping Teng

Krishnansu Tewari

Pham Quoc Thanh

Kamala Thriemer

Yaohua Tian

Zhang Tiejun

Kelvin To

Thaddäus Tönnies

Fatemeh Torabi

David Tordrup

Monica C Torres

Adrian Traeger

Annalisa Trama

Alexander Tran

Nguyen Thi Thu Trang

Tim Tsang

Agis Tsouros

Takeya Tsutsumi

Bayasgalan Tumenbayar

Gilad Twig

Gregory Tyrrell

Yutaka Ueda

Zulvikar Syambani Ulhaq

Wataru Umishio

Leanne Unicomb

Akihiko Usui

Ehsan Vaghefi

Tracy Vaillancourt

Alexandre Vallée

Victor van den Berg

Nick van Es

Edwin van Leeuwen

Ard van Sighem

Mandira Varma-Basil

Konstantina Vasilakopoulou

Andrey Vasin

Adam Vaughan

Arun Kumar Verma

Dominique Vervoort

Timo Vesikari

Federico Viganego

Nicolas Vignier

Lakshmi Vijayakumar

Joshua Vogel

Florian Vogt

Thi Thanh Hoa Vu

Abram Wagner

Timothy Walker

Marjan Walli-Attaei

Xia Wan

Yanmin Wan

Marta Wanat

Chi Chiu Wang

Chongjian Wang

Debin Wang

Haikun Wang

Hao-Yu Wang

Hongxing Wang

Huali Wang

Jiahao Wang

Jiajia Wang

Jian Wang

Jijun Wang

Lan Wang

Limin Wang

Ling Wang

Man Ping Wang

Shaobin Wang

Shibin Wang

Siqin Wang

Wei Wang

Yang Wang

Yaohui Wang

Yuan Yuan Wang

Zengwu Wang

Zhijie Wang

Zhilian Wang

Zhonglei Wang

Ahmed Waqas

Jeremy Ward

Leonor Mercedes Ward

Grant Waterer

Jianrui Wei

Jie Wei

Jingkai Wei

Xiang Wei

Yongyue Wei

Anna-Karin Welmer

Jianping Weng

David Whiteman

Martin Whyte

Anna Wilkinson

Ruben Willems

Rebecca Winter

Frank Wong

Martin Wong

Paulina Wong

Robert Wong

William W L Wong

Xin Ci Wong

Jean Woo

James Wood

Mark Wooden

Orlando Woods

Chenkai Wu

Chunmei Wu

Fang Wu

Jaw-Ching Wu

Jiayuan Wu

Liyun Wu

Rui Wu

Simiao Wu

Tongning Wu

Anne Wyllie

Grant Wyper

Casey Xavier Hall

Yu-Tao Xiang

Hong Xiao

Yonghong Xiao

Yu Xiao

Yue Xie

Liying Xing

Xi Xiong

Kai-Feng Xu

Shunqing Xu

Xiaolin Xu

Pragya Yadav

Rakesh Yadav

Jing Yan

Jingjing Yan

Lijing Yan

Wenhua Yan

Zhipeng Yan

Bingyi Yang

Gonghuan Yang

Jun Yang

Li Yang

Liyan Yang

Lu Yang

Ming Yang

Xiaoming Yang

Yonghong Yang

Yue Yang

Kai-hu Yao

Marie Yap

Hadi Yassine

Li Ye

Shuang Ye

Hung-I Yeh

Eng-Kiong Yeoh

Aiwen Yi

Honglei Yin

Peng Yin

Wenjin Yin

Zundong Yin

Paul Yip

Lay Myint Yoshida

Hiroshi Yoshino

Eiji Yoshioka

Paul Young

Yiming Yu

Kwok-Yung Yuen

Amanda Yufika

Neily Zakiyah

Dorota Zarębska-Michaluk

Mohammad Javad ZareSakhvidi

Jan Maciej Zaucha

Seraphine Zeitouny

Cherri Zhang

Chi Zhang

Fengqing Zhang

Hongxia Zhang

Jun Zhang

Jun Zhang

Lei Zhang

Luwen Zhang

Qingpeng Zhang

Yanchun Zhang

Yingchun Zhang

Yuan Zhang

Yong Zhao

Danying Zheng

Jialin Zheng

Zhi-Jie Zheng

Jian Zhou

Xiao-Nong Zhou

Xinfa Zhou

Yi-Hua Zhou

Ying Zhou

Jingfen Zhu

Yifan Zhu

Guihua Zhuang

Xiantong Zou

Zhuoru Zou

